# Dural tear of unusual cause

**DOI:** 10.11604/pamj.2015.20.189.6175

**Published:** 2015-02-27

**Authors:** Hicham Kechna, Jaouad Loutid, Omar Ouzzad, Sidi Mohamed Hanafi, Moulay Ahmed Hachimi

**Affiliations:** 1 Anesthesia Resuscitation Pole- Military Hospital Meknes, Morroco

**Keywords:** Headache, spinal analgesia, epidural anaesthesia, post-dural puncture headache

## Abstract

Epidural analgesia is highly recommended in cancer anorectal surgery. In addition to the fight against pain it provides some benefit in allowing early rehabilitation of patients. One of the risks of this practice is the dural tear creating a cerebrospinal fluid leak (CSF) in the epidural space (EPD). Clinical features the typical positional headache, a procession of various more or less severe symptoms: nausea, vomiting, dizziness, visual or hearing impairment or radicular pain. We report a dural of unusual cause secondary of the obstruction of tuohy catheter by vertebral cartilage.

## Introduction

The lumbar post puncture syndrome (LPPS) occurs after a break duro subarachnoid during an epidural puncture creating a cerebrospinal fluid leak (CSF) in the epidural space (EPD). Clinical features the typical positional headache, a procession of various more or less severe symptoms: nausea, vomiting, dizziness, visual or hearing disturbances or radicular pain. The occurrences of circumstances are numerous, we report the case of a dural breach occurred when performing epidural analgesia for digestive cancer surgery of an unusual cause.

## Patient and observation

**Figure 1:**
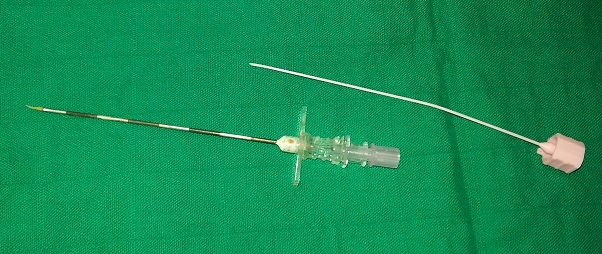
Images showing the tip of cartilage responsible for the obstruction of the epidural catheter

It is a 46 year old patient without significant medical history and normal weight, admitted to visceral surgery block for sigmoid tumor resection. Epidural analgesia was used for the management of perioperative pain. After installation and commissioning requirement in this kind of anesthetic practice, the realization of the puncture was at L2-L3 by an experienced anesthetist. After introducing the catheter 2-3 cm and removing the plastic core research epidural space using against fluid pressure was started. Bone obstacle was felt and the needle was redirected in a cephalic direction. To the failure of catheterization despite the introduction of the needle tuohy over its entire length, the latter was withdrawn for verification. It was then that we discovered the obstruction of the catheter by a piece of bone cartilage (Figure 1) dural tear was strongly suspected despite the non completion of cerebrospinal fluid through the needle. The second attempt was successful at the underlying spaced. The immediate postoperative analgesia was based on an intravenous analgesics and the epidural wasn't started until fully awake and lucid contact with the patient under the close supervision of an anesthesic nurse care even after returning to service. However, the day after surgery, the patient complained of typical post-dural puncture syndrome what motivated the execution of a blood patch. The evolution was very favorable.

## Discussion

The dural tear is one of the most common complications of locating the epidural space. The operator experience is a commonly recognized risk factor. so, if a frequency of about 1% is generally accepted in the research of the lumbar epidural space [1], Norris et al. [2, 3] have reveal that the frequency can reach 2.7% when the majority of acts practiced by internal. The role of patient characteristics making it difficult to find the epidural space, especially obesity, is less straightforward. Indeed, three-quarters of cases of dural breaches occurred in the first or second attempt at identification. However, it was recently shown that the period of custody is an additional risk factor in the development of this complication. [4] After a dural tear, the majority of anesthesiologists, as was the case in our observation, search the epidural space in an intervertebral spacing or sus- underlying for epidural analgesia [5, 6]. However, it should be emphasized that epidural catheter should be used with caution. Indeed, a significant portion of the solution administered into the epidural space can pass into the subarachnoid space and promote the expansion of the block [7, 8], with, in the extreme, the risk of total spinal anesthesia [9]. This passage is particularly important that the breach was made with a needle of large diameter [10], especially with a needle of 17 gauge. It is for this reason that epidural analgesia for our patient was started that after a complete awakening and under close supervision. Headache is the main result of the dural tear. Their frequency is 30 to 70% of cases of proven breach [11], which can often be more important in obstetric epidural analgesia due to the age of patients, the large diameter of the needle used for marking the epidural space and the role favoring any thrust forces in the expulsion phase [12]. For some teams any postural headache in the aftermath of epidural analgesia should be considered and treated as a MPPC. [13] While many therapeutic approaches are proposed (rest, establishment of a lap belt, hydration, analgesics, caffeine, sumatriptan, epidural infusion of crystalloid or colloid) [6, 14] the radical cure of PDPH is always based on achieving a epidural blood patch because the severity and nature of these debilitating headaches quickly leads to depressive manifestations of women and aggressiveness of the environment. In our case, being the circumstances of the execution, the diagnosis of dural tear was quickly adopted and bood patch immediately realized. However, for the case of dural tear proved with continued epidural analgesia using a catheter placed in a space or sus- underlying the realization of a blood preventive patch by the epidural catheter may be considered. Indeed, several studies show that preventive blood patch greatly decreases the frequency and intensity of CPPD probably [15, 16]. In our case the tear was strongly suspected and the blood patch made the first complaint headaches. The incidence of epidural blood patch success of around 90%, which means that about 10% of patients had residual headaches after blood patch. In these cases, a second blood patch should be offered.

## Conclusion

After accidental dural tear, analgesia may be continued by epidural (obstetrics). In case of epidural analgesia, a prophylactic blood patch seems desirable. This observation highlights the importance of greater caution in the progression of the needle tuohy and the verification of permeability of the needle every each bone contact.
